# What Do You Think You Are Measuring? A Mixed-Methods Procedure for Assessing the Content Validity of Test Items and Theory-Based Scaling

**DOI:** 10.3389/fpsyg.2017.00126

**Published:** 2017-02-21

**Authors:** Ingrid Koller, Michael R. Levenson, Judith Glück

**Affiliations:** ^1^Department of Psychology, Alpen-Adria-Universität KlagenfurtKlagenfurt, Austria; ^2^Department of Human Development and Family Sciences, Oregon State UniversityCorvallis, OR, USA

**Keywords:** content validity, mixed-methods, CSS-procedure, wisdom, item response models, partial credit model, theory-based item analysis, Adult Self-Transcendence Inventar (ASTI)

## Abstract

The valid measurement of latent constructs is crucial for psychological research. Here, we present a mixed-methods procedure for improving the precision of construct definitions, determining the content validity of items, evaluating the representativeness of items for the target construct, generating test items, and analyzing items on a theoretical basis. To illustrate the mixed-methods content-scaling-structure (CSS) procedure, we analyze the Adult Self-Transcendence Inventory, a self-report measure of wisdom (ASTI, Levenson et al., 2005). A content-validity analysis of the ASTI items was used as the basis of psychometric analyses using multidimensional item response models (*N* = 1215). We found that the new procedure produced important suggestions concerning five subdimensions of the ASTI that were not identifiable using exploratory methods. The study shows that the application of the suggested procedure leads to a deeper understanding of latent constructs. It also demonstrates the advantages of theory-based item analysis.

## Introduction

Construct validity is an important criterion of measurement validity. Broadly put, a scale or test is valid if it exhibits good psychometric properties (e.g., unidimensionality) and measures what it is intended to measure (e.g., Haynes et al., 1995; de Von et al., 2007). The 2014 Standards for Educational and Psychological Testing (American Educational Research Association, American Psychological Association, and National Council on Measurement in Education, 2014) state that test content is one of four interrelated sources of validity, the other ones being internal structure, response processes, and relations to other constructs. While elaborate empirical and statistical procedures exist for evaluating at least internal structure and relations to other constructs, the validity of test content is harder to ensure and to quantify. As Webb (2006) wrote in his chapter in the Handbook of Test Development, “Identifying the content of a test designed to measure students' content knowledge and skills is as much an art as it is a science. The science of content specification draws on conceptual frameworks, mathematical models, and replicable procedures. The art of content specification is based on expert judgments, writing effective test items, and balancing the many tradeoffs that have to be made” (p. 155). This may be even more the case when the construct at hand is not a form of knowledge or skill, where responses can be coded as right or wrong, but a personality or attitudinal construct where responses are self-judgments. How can we evaluate the validity of the items that form a test or questionnaire?

The development and evaluation of tests and questionnaires is a complex and lengthy process. The phases of this process have been described, for example, in the Standards for Educational and Psychological Testing by the American Educational Research Association, American Psychological Association, and National Council on Measurement in Education (2014), or by the Educational Testing Service (2014). A good overview is given by Lane et al. (2016). These standards require, for example, that the procedures for selecting experts and collecting their judgments should be fully described, or that the potential for effects of construct-irrelevant characteristics on the measure should be minimized. Here, we describe a concrete procedure for evaluating and optimizing the content validity of existing and new measures in a theory-based way that is consistent with the requirements of the various standards. That is, we do not focus on ways to develop new test items in a theory-consistent way, which are described at length in the literature (e.g., Haladyna and Rodriguez, 2013; Lane et al., 2016), but on how to evaluate existing items in an optimal, unbiased way.

Content validity (Rossiter, 2008) is defined as “the degree to which elements of an assessment instrument are relevant to a representative of the targeted construct for a particular assessment purpose” (Haynes et al., 1995, p. 238). Content validity includes several aspects, e.g., the validity and representativeness of the definition of the construct, the clarity of the instructions, linguistic aspects of the items (e.g., content, grammar), representativeness of the item pool, and the adequacy of the response format. The higher the content validity of a test, the more accurate is the measurement of the target construct (e.g., de Von et al., 2007). While we all know how important content validity is, it tends to receive little attention in assessment practice and research (e.g., Rossiter, 2008; Johnston et al., 2014). In many cases, test developers assume that content validity is represented by the theoretical definition of the construct, or they do not discuss content validity at all. At the same time, content validity is a necessary condition for other aspects of construct validity. A test or scale that does not really cover the content of the construct it intends to measure will not be related to other constructs or criteria in the way that would be expected for the respective construct.

In a seminal paper, Haynes et al. (1995) emphasized the importance of content validity and gave an overview of methods to assess it. After the publication of this paper, consideration of content validity in assessment studies increased for a short time, especially in the journal where the authors published their work. However, a brief literature search in journals relevant to the topic shows that content validity is still rarely referred to and even less often analyzed systematically. Between 1995 and 2015, “content validity” was mentioned in one article published in *Assessment*, in 44 articles in *Psychological Assessment*, where the paper by Haynes et al. (1995) was published, in 22 articles in *Educational Assessment*, and in seven articles in *European Journal of Psychological Assessment*. Currently, content validity is rarely mentioned in psychological journals but receives special attention in other disciplines such as nursing research (e.g., de Von et al., 2007).

### Methods to evaluate content validity

Several approaches to evaluate content validity have been described in the literature. One of the first procedures was probably the Delphi method, which was used since 1940 by the National Aeronautics and Space Administration (NASA) as a systematic method for technical predictions (see Sackman, 1974; Hasson and Keeney, 2011). The Delphi method, which is predominantly used in medical research, is a structural iterative communication technique where experts assess the importance of characteristics, symptoms, or items for a target construct (e.g., Jobst et al., 2013; Kikukawa et al., 2014). In the first round, each expert individually rates the importance of symptoms/items for the illness/construct of interest. In the second round, the experts receive summarized results based on the first round and can make further comments or revise their answers of the first round. The process stops when a previously defined homogeneity criterion is achieved.

Most procedures currently used to investigate content validity are based on the quantitative methods described by Lawshe (1975) or Lynn (1986), who also provided numerical content validity indices (see Sireci, 1998). All these methods, as well as the Delphi-method, are based on expert judgments where a number of experts rate the relevance of the items for the construct on 4- to 10-point scales or using percentages (Haynes et al., 1995) or assign the items to the dimensions of the construct (see Moore and Benbasat, 2014). Then, indicators of average relevance are calculated. This can be done by calculating simple average percentages (e.g., if the number of experts is low) or by using a cut-off value (usually 70–80%) to decide whether an item measures the respective construct (e.g., Sireci, 1998; Newman et al., 2013). In other cases, as mentioned above, a content validity index (Lawshe, 1975; Lynn, 1986; Polit and Beck, 2006; Polit et al., 2007; Zamanzadeh et al., 2014) is estimated for individual items (e.g., the proportion of agreement among experts concerning scale assignment) or for the whole scale (e.g., the proportion of items that are judged as content valid by all experts).

There exists, however, no systematical procedure that could be used as a general guideline for the evaluation of content validity (cf. Newman et al., 2013). Also, Johnston et al. (2014) recently called for a satisfactory, transparent, and systematical method to assess content validity. They described a quantitative “discriminant content validity” (DCV) approach that assesses not only the relevance of items for the construct, but also whether discriminant constructs play an important role for response behavior. In this method, experts evaluate how relevant each item is for the construct. After that, it is statistically determined whether each item measures the construct of interest. This procedure is well-suited for determining content validity, but the authors argued that it is not possible to determine the representativeness of the items for the target construct, as claimed by Haynes et al. (1995). Furthermore, it involves only purely quantitative analyses and does not utilize the potential of qualitative approaches. For example, Newman et al. (2013) illustrated the advantages of mixed-method procedures for evaluating content validity or even validity in general. They specifically mention two advantages: the possibility to improve validity and reliability of the instrument, and the resulting new insights into the nature of the target construct. They introduced the Table of Specification (ToS), which requires experts, among other things, to assign items to constructs using percentages. Additionally, the experts can estimate the overall representativeness of the whole set of items for the target construct and add comments concerning this topic. The ToS is wide applicable and a good possibility to evaluate content validity, but it does not allow the evaluation of overlaps between different constructs and does not connect the results to the subsequent psychometric analysis of items. Such a connection does not only improve the measurement of content validity, it also allows for theory-based item analysis. That is, expert judgments about item content can be used to derive specific hypotheses about item fit, which can then be tested statistically.

### Theory-based item analysis

Scale items are often developed on the basis of theoretical definitions of the construct, and sometimes they are even analyzed for content validity in similar ways as described above, but after this step, item selection is usually purely empirical. A set of items is completed by a sample of participants, and response frequencies and indicators of reliability such as item-total correlations are used to select the best-functioning items. Rossiter (2008) criticized that at the end of such purely empirical item-selection processes, the remaining items often measure only one narrow aspect of the target construct. In such cases, it would perhaps be possible to retain the diversity of the original items by constructing subscales. Some authors, such as Gittler (1986) and Koller et al. (2012), have long highlighted the importance of theory-based analysis. For example, Koller and Lamm (2015) showed that a theory-based analysis of items measuring cognitive empathy yielded important information concerning scale dimensionality. In this study, the authors derived hypotheses about item-specific violations of the Rasch model from expert judgments about item content. The expert judgements suggested that the perspective-taking subscale could theoretically be subdivided into the dimensions “to understand both sides” and “to put oneself in the position of another person.” Psychometric analysis confirmed this assumption, which is also in accordance with recent findings from social neuroscience (e.g., Singer and Lamm, 2009). In sum, approaches from neuropsychology, item response theory, and qualitative item-content analysis were integrated into a more valid assessment of cognitive empathy.

### Goals of the study

The main aim of this paper is to introduce the *Content-Scaling-Structure* (CSS) procedure, a mixed-methods approach for analyzing and optimizing the content validity of a questionnaire or scale. The approach is suitable for many research questions in the realm of content validity, such as the formulation of a-priori hypotheses for a theory-based item analysis, the refinement of the definition of a target construct, including possible differentiation into subdimensions, the evaluation of representativeness of items for the target construct, or the investigation of the influence of related constructs on the items of a scale. Thus, the proposed CSS procedure combines the qualitative and quantitative investigation of content validity with an approach for the theory-based psychometric analysis of items. Furthermore, it can lead to a better understanding of the latent construct itself, a better embedding of empirical findings in the research literature, and to a higher construct validity of the instrument in general. We present a general description of the procedure (see Table 1) and describe several possible adaptations. The proposed procedure can be adapted in several ways to examine different types of latent constructs (e.g., competencies vs. traits, less vs. more complex constructs). In summary, the proposed CSS procedure fulfills the demand for a systematical and transparent procedure for the estimation of content validity, includes the advantages of mixed-methodologies, and allows researchers not only to evaluate content validity, but also to perform theory-based item analyses. Although the CSS procedure was developed independently of the ToS (Newman et al., 2013), there are several similarities. However, the CSS-procedure is not limited to the non-psychometric analysis of content validity, it also includes psychometric analyses and the integration of the non-psychometric and psychometric parts. Accordingly, the first part of the CSS-procedure could be viewed as an adaption and extension of the ToS.

**Table 1:** **General schedule for the Content-Scaling-Structure (CSS) procedure**.

**Step**	**Description**
1. Development of the expert questionnaire	Define clear instructions and working definitions for the subdimensions of the target construct; construct an item booklet.
2. Selection of experts	Select a minimum of five experts from different fields (including experts from within and outside the respective content domain and experts in psychometrics).
3. Individual data collection with each expert	Face to face interview or survey study (paper-pencil, online); no time limit.
4. Summary of the results based on predefined rules	Summarize the results: mean percentages of the assignments, relevant dimensions for each item. Content-analyze responses to open-ended questions or think-aloud responses.
5. Meeting of the experts, discussion of the results	A minimum of two experts from different fields discuss the results (optimally, all experts in a focus group setting). Possibly: second round of individual assignment of the items to dimensions.
6a. Final assignment of the items to the dimensions	In a second discussion with the experts, finalize the assignment of items to dimensions, modify the original dimension definitions, taking into account the theoretical and empirical literature.
6b. Definition of possible psychometric hypotheses	Define psychometric hypotheses (e.g., dimensionality) and psychometric problems (e.g., DIF, comprehension problems).
6c. Definition of possible associations between dimensions	If possible/desirable, define different structural models for the instrument (e.g., unidimensional vs. multidimensional).
7. Validation study	Investigate the validity of the instrument in a representative sample using an appropriate psychometric model (item-response models, factor-analytic approaches).
8. Final definition of the latent construct. If necessary go back to point 1 or to point 5	Based on all results, refine the operational definition of the target construct measured by the instrument, and identify other latent constructs that influence the response process. Based on the research interest, answer further questions to topics like discriminant and congruent validity, representativeness of the items for the target construct, or integrate the results in the state of the research of the target construct.

To demonstrate our procedure here, we use the Adult Self-Transcendence Inventory (ASTI), a self-report scale measuring the complex target construct of wisdom. In the first part of the study, an expert panel analyzed the content of the ASTI items with respect to the underlying constructs in order to investigate dimensionality, identify potential predictors of differential item functioning, and analyze the appropriateness of the definition of the construct for the questionnaire. In the second part, data from a sample of 1215 participants were used to evaluate the items using multidimensional item response theory models, building upon the results of the first part. It is not at all mandatory to use item response modeling for such analyses; other psychometric methods, such as exploratory or confirmatory factor analyses, may also be employed, although our impression is that item response models are particularly well-suited to test specific hypotheses about item functioning. At the end, the results and the proposed procedure are discussed and a new definition of the target construct that the ASTI measures is given.

Before we describe the CSS-procedure in detail, we introduce the topic of measuring wisdom and the research questions for the presented study. After that, the CSS procedure is described and illustrated using the ASTI as an example.

### Measuring wisdom with the adult self-transcendence inventory

Wisdom is a complex and multifaceted construct, and after 30 years of psychological wisdom research, measuring it in a reliable and valid way is still a major challenge (Glück et al., 2013). In this study, we used the ASTI, a self-report measure that conceptualizes wisdom as self-transcendence (Levenson et al., 2005). The idea that self-transcendence is at the core of wisdom was first brought forth by the philosopher Trevor Curnow (1999) in an analysis of the common elements of wisdom conceptions across different cultures. Curnow identified four general principles of wisdom: self-knowledge, detachment, integration, and self-transcendence. Levenson et al. (2005) suggested to consider these principles as stages in the development of wisdom.

*Self-knowledge* is awareness of the sources of one's sense of self. The sense of self arises in the context of roles, achievements, relationships, and beliefs. Individuals high in self-knowledge have developed awareness of their own thoughts and feelings, attitudes, and motivations. They are also aware of aspects that do not agree with their ideal of who they would like to be.

Individuals high in *non-attachment* are aware of the transience and provisional nature of the external things, relationships, roles, and achievements that contribute to our sense of self. They know that these things are not essential parts of the self but observable, passing phenomena. This does not mean that they do not care for their relationships—on the contrary, non-attachment increases openness to and acceptance of others: individuals who are less identified with their own wishes, motives, thoughts, and feelings are better able to perceive, and care about, the needs and feelings of others.

*Integration* is the dissolution of separate “inner selves.” Different, contradictory self-representations or motives are no longer in conflict with each other or with the person's ideal self, but accepted as part of the person. This means that defense mechanisms that protect self-worth against threats from undesirable self-aspects are no longer needed.

*Self-transcendent* individuals are detached from external definitions of the self. They know and accept who they are and therefore do not need to focus on self-enhancement in their interaction with others. For this reason, they are able to dissolve rigid boundaries between themselves and others, truly care about others, and feel that they are part of a greater whole. Levenson et al. (2005) argue that self-transcendence in this sense is at the core of wisdom.

Levenson et al. (2005) developed a first version of the ASTI to measure these four dimensions. That original version consisted of 18 items with a response scale that asked for changes over the last 5 years rather than for participants' current status. This approach turned out to be suboptimal, however, and a revised version was developed that consists of 34 items with a more common response scale ranging from 1 (“disagree strongly”) to 4 (“agree strongly”). Some items were reworded from Tornstam's (1994) gerotranscendence scale, others were newly constructed by the test authors in order to broaden the construct. The majority of the items are related to one of the four dimensions identified by Curnow (1999). They refer to inner peace independent of external things, feelings of unity with others and nature, joy in life, and an integrated sense of self. Ten of the items were included to measure alienation as a potential opposite of wisdom.

In a study including wisdom nominees as well as control participants, Glück et al. (2013) used a German-language version of the revised ASTI. The scale was translated into German and back-translated. One of the items (“Whatever I do to others, I do to myself”) was difficult to translate into German because it could have two different meanings (item 34: doing something (good) for others and oneself, or item 35: doing something (bad) to others and oneself). After consultation with the scale author, M. R. Levenson, both translations were retained in the German scale, resulting in a total of 35 items. In study by Glück et al. (2013), the ASTI had the highest amount of shared variance with three other measures of wisdom, which suggests that it may indeed tap core aspects of wisdom. Reliability was satisfactory (Cronbach's α = 0.83), but factor analyses using promax rotation suggested that the ASTI might be measuring more than one dimension. The factor loadings did not suggest a clear structure representing Curnow's four dimensions, however. Scree plots suggested either one factor (accounting for only 21.7% of the variance) or three factors (38.5%). The first factor in the three-factor solution comprised five items describing the factor of self-transcendence (feeling one with nature, feeling as part of a greater whole, engaging in quiet contemplation), but the other two factors included only two or three items each and did not really correspond with the subfacets proposed by the scale authors. Thus, factor-analytically, the structure of the ASTI was not very clear. As the scale had not been systematically constructed to include specific subscales, there was no basis for a confirmatory factor analysis, and given the exploratory results it seems doubtful that such an analysis would have rendered clear results.

In the current study, we used the CSS procedure to gain insights about the structure of the scale, with the goal of identifying possible subscales. We only analyzed the 24 items measuring self-transcendence, excluding the 10 alienation items. Tables **3A–C** include all analyzed items. Before the point by point description of the CSS Procedure, the research questions for the investigation of the ASTI are described.

## Description and application of the content-scaling-structure procedure

As the study design is intended to be a template for other studies, we first present a point-by-point description of the steps in Table 1. In general, detailed descriptions of the procedure for assigning items to scales are important elements for the transparency of studies, especially as they offer the possibility to reanalyze and compare results across different studies (e.g., Johnston et al., 2014).

The research questions for the current study were as follows.

- *Which items of the ASTI fulfill the requirement of content validity?* The theoretical background of the ASTI describes a multidimensional, complex construct, and it is not obvious for all items to which dimension(s) they pertain. In addition, item responses may be influenced by other, related constructs that the ASTI is not intended to tap. Thus, we investigated experts' views on the relation of the items to the dimensions of the ASTI and to related constructs. The results of this analysis were also expected to show whether all dimensions of the ASTI are well-represented by the items, i.e., whether enough items are included to assess each dimension.
- *How sufficient is the definition of the four-factor model underlying the ASTI?* In earlier studies using content-dimensionality scaling, we have repeatedly found that the exercise of assigning items to dimensions and discussing our assignments has enabled us to rethink the definitions of the constructs themselves, identify conceptual overlaps between dimensions, and find that certain aspects were missing in a definition or did not fit into it. In other cases, we found that a construct was too broadly defined and needed to be divided into sub-constructs. In this vein, it is also important to consider whether other, related constructs may affect the responses to some items.
- *Do the expert judgments suggest hypotheses for theory-based item analysis?* The expert judgments are interesting not only with respect to the dimensionality of the ASTI. Other relevant psychometric issues include potential predictors of differential item functioning, comprehensibility of items, and too strong or too imprecise item formulations.
- *To what extent are the theory-based assignments of items to dimensions supported by psychometric analyses?* The ultimate goal of the study was to identify unidimensional subscales of the ASTI that would include as many of the items as possible and make theoretical as well as empirical sense. In earlier studies, we have repeatedly found that subscales that were identified in a theory-based way remained highly stable in cross-validations with independent samples, whereas subscales determined in a purely empirical way did not.

### Development of the expert questionnaire: instruction, definitions, and item booklet

Independent of whether the research question of interest concerns the construction of a new measurement instrument, the evaluation of an existing measurement, or evaluating the representativeness of the items for the target construct, the first step is to lay out the definition of the target construct in a sufficiently comprehensive way (e.g., by a systematical review). That is, all relevant dimensions of the target construct should be defined in such a way that there is no conceptual overlap between them. Because the goal of the current study was to investigate the items and definitions of the ASTI and not the representativeness of the items for the construct of wisdom, we used the four levels of self-transcendence (self-knowledge, non-attachment, integration, and self-transcendence) as defined above.

**Figure 1:**
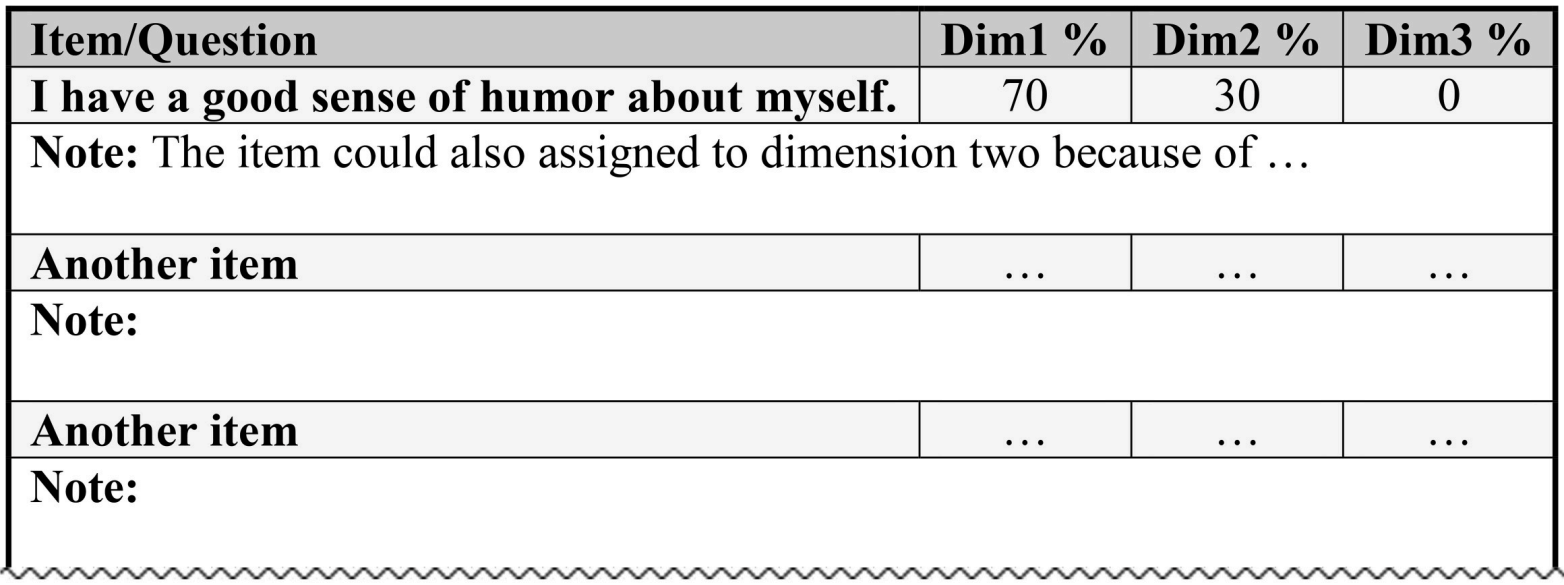
**A fictive example of an item-booklet**.

The second step is the generation of the questionnaire for the expert ratings based on the definitions of the target dimensions and the items. It includes a clear written instruction, the definitions of each subdimension, and an item booklet (see Figure 1) with the items in random order (i.e., not ordered by subdimension or content).

In the present study, the experts were first instructed to carefully read the definitions of the four dimensions underlying the ASTI shown above. Then they should read each item and use percentages to assign each item to the four dimensions: if an item tapped just one dimension, they should write “100%” into the respective column, if an item tapped more than one dimension they should split the 100% accordingly. For example, an item might be judged as measuring 80% self-transcendence and 20% integration. It is also possible to “force” experts to assign each item to only one dimension. This might be useful for re-evaluating an item assignment produced in earlier CSS rounds. As a first step, however, we believe that this kind of assignment could lead to a loss of valuable psychometric information about the items and increase the possibility of assignment errors.

If the experts felt that an item was largely measuring something other than the four dimensions, they should not fill out the percentages but make a note in an empty space below each item. They were also asked to note down any other thoughts or comments in that space. In addition, they were asked two specific questions: (1) “Do you think the item could be difficult to understand? If yes, why?,” and (2) “Do you think the item might have a different meaning for certain groups of people (e.g., men vs. women, younger vs. older participants, participants from different professional fields, or levels of education)? If yes, why?” The responses to these last questions allowed us to formulate a-priori hypotheses about differential item functioning (DIF; e.g., Holland and Wainer, 1993; see Section Testing Model Fit).

### Selection of experts

We recommend to recruit at least five experts (cf. Haynes et al., 1995): (1) at least two individuals with high levels of scientific and/or applied knowledge and experience concerning the construct of interest and related constructs, and (2) at least two individuals with expertise in test construction and psychometrics, who evaluate the items from a different perspective than content experts, so that their answers may be particularly helpful for theory-based item analysis. In addition, depending on the construct of interest, it may be useful to include laypersons, especially individuals from the target population (e.g., patients for a measure of a psychiatric construct). A higher number of experts allows for a better evaluation of the consistency of their judgments, increases the reliability and validity of the results, and allows the calculation of content validity indices. However, a higher number of experts can also increase the complexity of the results. In any case, the quality and diversity of the experts may be more important than their number.

In the present study, nine experts (seven women and two men) participated. Because the interest of the study was to validate the items of the ASTI, all experts were psychologists; five were mainly experts in the field of wisdom research and related fields (including the second and third author), and four were mainly experts of test psychology and assessment in different research fields (including the first author). All experts worked with the German version of the ASTI, except for the second author who used the original English version. In the present study, the experts were invited by email and the questionnaire was sent to them as an RTF document.

### Individual data collection with each expert

The experts filled out the questionnaire individually and without time limits.

### Summary of the results based on predefined rules

Next, the responses of the experts were summarized according to pre-defined rules. As Table 2 shows, we produced one summarizing table for each item. In this table, the percentages provided by the experts were averaged. In addition, we counted how many experts assigned each item to each dimension. Finally, the notes provided by the experts were sorted into three categories: notes concerning dimension assignment, item or dimension content, and psychometric issues.

**Table 2:** **Example of summarized results for the discussion of final assignments**.

**Expert**	***I*_1_*d*_1_**	***I*_1_*d*_2_**	***I*_1_*d*_3_**	**D_class**	**Note_D**	**Note_C**	**Note_P**
E1	70	30	0	1	Notes	…	…
E2	50	50	0	0	Notes	…	…
…	100	0	0	1	…	…	…
…	…	…	…	…	Notes	…	…
E*n*	…	…	…	…	Notes	…	…
*M*%	80	20	5				

*I_1_d_1_ means that item 1 is assigned to dimension 1; D_class is the dimension classification (e.g., 0 = undetermined, 1 = dimension 1, etc.); Note_D is for notes relating to the classification in dimensions; Note_C is for notes relating to content of item or construct, e.g.; the content of the item includes two different dimensions; Note_P is for notes to possible psychometric problems, e.g., too strong formulation of items; M% is the mean percentage per column*.

Of course, these three categories are only examples, other categories are also possible. This qualitative part of the study can offer theoretical insights about the target construct as well as the individual items.

The last row of Table 2 contains the average percentages for each dimension across all experts. These values allow for first insights concerning dimension assignments. For example, the mean values might be *d*_1_ = 50%, *d*_2_ = 30%, *d*_3_ = 10%, *d*_4_ = 10%. A cutoff value can be used to determine which dimensions are important concerning the item. Choice of the cutoff value depends on the research aims: if the goal is to select items that measure only one subcomponent of a clearly and narrowly defined construct, a cutoff of 80% may be useful. This would mean that the experts agree substantially that each item very clearly taps one and only one of the dimensions involved. Newman et al. (2013), for example, wrote that 80% agreement among experts is a typical rule of thumb for sufficient content validity. If the goal is to inspect the functioning of a scale measuring a broader, more vaguely defined construct, lower cutoff values may be useful. In addition to setting a cutoff, it may also be important to define rules for dealing with items that have relevant percentages on more than one subdimension. Our experience is that simply discarding all such items may mean discarding important aspects of the construct. It may be more helpful to consider alternative ways of dividing the scale into subcomponents. Even if an item has a high average assignment on only one dimension, it may still be worthwhile to check whether some experts assigned it strongly to another dimension. Thus, it is important to not only look at the averages, but to inspect the whole table for each item. In addition, if several rounds of expert evaluations are performed, the cutoff could be set higher from round to round.

In the current case, as the goal was not to select items but to gain information about an existing scale, we used a much lower cutoff criterion of 0.30 to determine which dimension(s) experts considered as most fitting for each item. We then presented the results, in an aggregated form, to a subgroup of the experts with the goal redefining and perhaps differentiating the dimensions to allow for a clearer assignment of items. Thus, the number of experts who assigned the item to the most prominent dimension with a percentage of at least the cutoff value was an indicator of homogeneity of the experts' views (see Tables 3A–C, column 4), similar to the classical content validity index (see Polit and Beck, 2006).

**Table 3A:** **Results of the final assignment of the ASTI items**.

**Nr**.	**Item**	**Mean % per assig Dim. (CI 95%)**	**Assignment in Dim. # ≥30%**	**Summarized comments**
**SELF-KNOWLEDGE AND INTEGRATION (SI)**				
I10	I have a good sense of humor about myself.	*m_*IN*_* = 36 (10.5–62.8)	IN = 5	In its earlier form (I don't take myself too seriously) the item didn't work.
		*m_*SK*_* = 18 (0.0-42.9)		
I19	I feel that I know myself.	*m_*SK*_* = 97 (89.0–100)	SK = 9	No comments.
I20	I am accepting of myself, including my faults.	*m_*IN*_* = 44 (18.1–70.8)	IN = 6	Different understanding of self-acceptance across different cultures.
		*m_*SK*_* = 40 (11.1–67.9)	SK = 4	
I21	I am able to integrate the different aspects of my life.	*m_*IN*_* = 86 (59.7–100)	IN = 8	Dependent on age and life situation.
**PEACE OF MIND (PM)**				
I01	I often engage in quiet contemplation.	*m_*NA*_* = (10.9–69.1)	NA = 4	It is more a component of emotion regulation; difficulties in comprehension.
I05	My peace of mind is not easily upset.	*m_*NA*_* = 29 (9.8–48.0)	NA = 4	Not possible to assign it to one dimension; the definition may be too imprecise; what does peace of mind mean?; life events could play a role.
I09	I do not become angry easily.	*m_*NA*_* = 38 (13.7–62.9)	NA = 5	Not possible to assign it to one dimension; the definition may be too imprecise; it is more a component of emotion regulation.
		*m_*SK*_* = 34 (4.5–64.4)	SK = 4	
I22	I can accept the impermanence of things.	*m_*NA*_* = 70 (41.8–97.1)	NA = 8	If a participant encountered a loss recently the item may be biased (emotion).

*Final Dim., final assigned dimension; mean % per assig Dim. (CI 95 = %), the mean percentages of the most relevant assignment and the 95% confidence interval of experts' assignments; Assignment in Dim. # ≥30%, the number of assignments above 0.30 by experts*.

**Table 3B:** **Results of the final assignment of the ASTI items**.

**Nr**.	**Item**	**Mean % per assign Dim. (CI 95%)**	**Assignment in Dim. # ≥30%**	**Summarized comments**
**NON-ATTACHMENT (NA)**				
I03	I don't worry about other people's opinions of me.	*m_*NA*_* = 44 (12.7–75.1)	NA = 5	Extraversion and egoisms can also play an important role.
		*m_*ST*_* = 33 (0.8–64.8)	ST = 3	
I06	My sense of well-being does not depend on a busy social life.	*m_*NA*_* = 51 (17.1–85.1)	NA = 5	Extraversion and egoisms can also play an important role; it could be easier if “social life” were replaced by “people.”
		*m_*ST*_* = 29 (0.2–58.0)	ST = 4	
I08	My happiness is not dependent on other people and things.	*m_*NA*_* = 42 (10.3–74.1)	NA = 5	Egoisms can also play an important role, difficult for so many postmodern people for whom “relationships” and possessions are paramount.
		*m_*ST*_* = 40 (11.0–69.0)	ST = 5	
I12	Material possessions don't mean much to me.	*m_*NA*_* = 58 (39.3–86.2)	NA = 7	Meaning depends on participant's material possessions.
		*m_*ST*_* = 32 (6.5–57.9)	ST = 5	
**SELF-TRANSCENDENCE (ST)**				
I02	I feel that my individual life is a part of a greater whole.	*m_*ST*_* = 75 (65.9–100)	ST = 8	Its dependent on the personal life situation, e.g., soldier.
I04	I feel a sense of belonging with both earlier and future generations.	*m_*ST*_* = 82 (56.2–100)	ST = 8	Dependent on age.
I07	I feel part of something greater than myself.	*m_*ST*_* = 87 (69.9–100)	ST = 9	Religiosity can play an important role.
I13	I feel compassionate even toward people who have been unkind to me.	*m_*ST*_* = 64 (33.6–93.1)	ST = 7	Empathy is an important component; the sentence is jolty; time lag can play a role (When was a person unfriendly to me?).
I16	I often have a sense of oneness with nature.	*m_*ST*_* = 69 (40.0–70.4)	ST = 7	Dependent on age; the absence of this sense is one of the most problematic issues in postmodern society.
I24	Whatever [good] I do for others, I do for myself.	*m_*ST*_* = 71 (20.0–100)	ST = 7	The understanding could be too Christian; dualists don't get it, it might be the only item the scale needs.
I25	Whatever [bad] I do to others, I do to myself.	*m_*ST*_* = 56 (13.7–97.7)	ST = 5	(German item) the understanding could be too Christian.

*Final Dim., final assigned dimension; mean % per assig Dim. (CI 95%), the mean percentages of the most relevant assignment and the 95% confidence interval of experts' assignments; Assignment in Dim. # ≥30%, the number of assignments above 0.30 by experts*.

**Table 3C:** **Results of the final assignment of the ASTI items**.

**Nr**.	**Item**	**mean % per assign Dim. (CI 95%)**	**Assignment in Dim. # ≥30%**	**Summarized comments**
**PRESENCE IN THE HERE-AND-NOW AND GROWTH (PG)**				
I11	I find much joy in life.	*m_*ST*_* = 33 (2.7–62.3)	ST = 3	In all four dimensions it is possible to have fun; not easy to assign it to one dimension; it is more a consequence of self-transcendence.
		*m_*SK*_* = 44 (3.9–83.6)	SK = 3	
I14	I am not often fearful.	*m_*ST*_* = 20 (0.0–39.6)	ST = 3	Not really possible to assign it to one dimension; it is a negatively formulated item; fearful about what?; the item works differently for women and men.
		*m_*SK*_* = 38 (2.1–74.6)	SK = 3	
I15	I can learn a lot from others.	*m_*ST*_* = 50 (16.3–83.7)	ST = 5	It is more a consequence of self-transcendence; dependent on situation.
		*m_*NA*_* = 34 (6.7–62.1)	NA = 4	
I17	I am able to accept my mortality.	*m_*ST*_* = 77 (56.1–98.4)	ST = 8	Dependent on situation (e.g., illness); based on the definition, it is not possible to assign it to one dimension, problematic for young and healthy people.
I18	I often “lose myself” in what I am doing.	*m_*ST*_* = 24 (0.0–56.6)	ST = 2	Flow item; it is more a consequence of self-transcendence.
		*m_*SK*_* = 36 (0.0–73.6)	SK = 4	
		*m_*NA*_* = 25 (0.0–63.7)	NA = 2	
I23	I have grown as a result of losses I have suffered.	*m_*ST*_* = 48 (s12.0–84.5)	ST = 4	Dependent on age, it is possible to grow in each dimension; it could be the path way to all dimensions.
		*m_*SK*_* = 22 (0.0–47.7)	SK = 2	
		*m_*NA*_* = 21 (0.1–41.2)	NA = 3	

*Final Dim., final assigned dimension; mean % per assig Dim. (CI 95%), the mean percentages of the most relevant assignment and the 95% confidence interval of experts' assignments; Assignment in Dim. # ≥30%, the number of assignments above 0.30 by experts*.

### Expert meeting: discussion of the results

Next, the experts are invited to discuss the assignments and comments as a group. This discussion is particularly fruitful if the assignments were relatively heterogeneous. It can lead to clarifications and possible modifications of the definitions of the dimensions, removal of items that clearly do not fit the construct, and even generation of additional items. If the original assignments were very heterogeneous, it makes sense to repeat the individual assignment and collective discussion in order to achieve a sufficient level of agreement among the experts. However, this iterative process can become very complex and is not always feasible. In any case, a minimum of two experts from different fields (for example, one content expert and one psychometrician) should make the decisions together.

The results of the analysis and discussion of the experts' assignments can take various forms. Usually, some items are clearly assigned to a specific dimension, others turn out to be so equivocal that they are eliminated. In some cases, however, the conceptualization of the dimensions needs to be reconsidered. For example, as mentioned above, if a number of items are assigned to two dimensions with about equal weight, this may mean that the two dimensions need to be collapsed or that an additional dimension is required that is conceptually located between the two. If the comments of experts provide new insights for possible dimension definitions or labels, these comments can also be included in the formulation of new definitions.

In the present study, it was not possible to discuss the results with all experts. Thus, the third author, an expert on the topic of wisdom and psychometrics, and the first author, a psychometrician not familiar with the concept of wisdom discussed the results, performed the final assignment of the items, and formulated new names and definitions for the resulting dimensions where they differed from the original ones.

### Final assignments, modified definitions, and possible associations between dimensions

#### Final assignment of the items to the dimensions

The results based on the assignments and the final discussion of the two experts are given in Tables 3A–C. Only important results are presented here. The third columns show only the final assignments to subdimensions, the fourth columns show only the relevant mean percentages and confidence intervals, and the fifth columns show the number of experts who assigned the item to one dimension with a minimum of 30% (homogeneity of expert judgments). The last columns present the summarized comments without any categorization because the number of comments was generally low. As the tables show, new dimensions such as “Peace of Mind” emerged in the discussion of the commonalities among items that seemed to tap an affective aspect of non-attachment. In the following, we describe the content of the scales that emerged from the final assignments and propose psychometric hypotheses for each subdimension.

##### “Self-knowledge and self-integration” (SI)

The four items in this scale all describe aspects of knowing and accepting oneself, including possibly diverging aspects and positive and negative sides (see Table 3A). Thus, the overarching theme of this subscale is knowing, accepting, and integrating the aspects of oneself and one's life. One item (“I feel that I know myself”) was theoretically assigned to the subdimension of self-knowledge only, the others were assigned to self-knowledge as well as integration or to integration only. The two subdimensions were merged into one scale based on the rationale that self-knowledge can be considered as a precondition for integration. Item 10 (“I have a good sense of humor about myself”) was problematic as it was assigned to a minimum of three categories. We eventually assigned it to “self-knowledge and integration” because it reflects a kind of benevolent acceptance of oneself including one's flaws, but made a note to specifically look at the fit of this item with the others in the psychometric analyses.

##### “Peace of mind” (PM)

All items of this scale are about valuing and maintaining one's tranquility even in the face of reasons to get angry or upset (see Table 3A). Item 22, “I can accept the impermanence of things,” seems to deviate somewhat from this pattern, but at closer inspection the ability to accept that all things are impermanent is about being able to remain calm in the face of losses as well as gains.

##### “Non-attachment” (NA)

This scale comprises items concerning the individual's independence of external things, namely, other people's opinions, a busy social life, or material possessions, and of other people and things in general (see Table 3B). Thus, it clearly corresponds to Curnow's (1999) concept of non-attachment. All items in this scale were predominantly assigned to the non-attachment component, but also, with percentages ranging from 29 to 40, to the self-transcendence component. This suggests that the experts considered the individual's independence of external sources of reinforcement as a part or precondition of self-transcendence. One reason may be that our definition of self-transcendence included the statement that self-transcendent individuals are detached from external definitions of self, which was based on the idea that self-transcendence is the last stage of a development through the other stages. As mentioned above, for the independent measurement of the four dimensions, it would seem important to avoid such conceptual overlaps. In any case, the common characteristic of the four items is their reference to non-attachment.

##### ‘Self-transcendence” (ST)

All items in this scale were unanimously assigned to the self-transcendence dimension; they refer to individuals experiencing themselves as part of or closely related to something larger than themselves—“a greater whole,” “earlier and future generations,” or nature (see Table 3B). Item 13, “I feel compassionate even toward people who have been unkind to me,” also suggests that the individual can relate to others on a general level that goes beyond personal relations. The essence of this scale is perhaps best captured by item 02, “I feel that my individual life is a part of a greater whole.”

##### “Presence in the here-and-now and growth” (PG)

The fifth dimension was labeled “presence in the here-and-now and growth”: its items describe individuals who are able to live in the moment: they find joy in their life and in what they are doing in a given moment, without being fearful of the future or preoccupied with the finitude of life (see Table 3C). They are aware that things are always changing, oriented toward learning from others, and aware that they have grown through losses.

#### Definition of psychometric hypotheses

A goal of the analyses was to test whether the hypotheses gained from the expert judgments could be used to improve the psychometric functioning of the ASTI. Specifically, we wanted to test whether the ASTI as a whole formed an unidimensional scale, and if not, whether the five subdimensions derived from the expert assignments of the items would form unidimensional scales. Also, we wanted to test whether single items within each scale diverged from the others. For the theory-based item analysis, we summarized the comments from Tables 3A–C into psychometric categories. These categories are not only useful for the interpretation of non-conforming items, but also for the construction of new additional items. We identified three main categories of expert comments:

- Test fairness (e.g., differential item functioning; see Section Testing Model Fit): For eight items (I02, I05, I12, I15, I17, I20, I21, I22), the experts noted possible context dependencies (e.g., the response may be dependent on life situation, life events, material possessions, health, or culture) and for five items (I04, I16, I17, I21, I23), possible influences of respondent age. For one item (I14), the experts suspected differences between men and women.
- Influences of other constructs: The expert judgments generally suggested that the items of the ASTI are good indicators for the target construct. But for some items, other constructs, such as emotion regulation (I01, I09), extraversion (I03, I06), egoism (I03, I06, I08), empathy (I13), or spirituality (I07, I24, I25) may influence responses.
- Linguistic factors: Only for five items (I01, I05, I06, I13, I14) linguistic problems (e.g., difficulties in comprehension) were suspected.

#### Definition of possible associations between dimensions

Sometimes researchers have theoretical assumptions about relationships between the various dimensions. Item response models can be used to test such hypotheses, e.g., to test predictions about correlations between dimensions or the structural relationships among them. In the current example, we only explored the latent correlations between the dimensions.

## Validation study

In the current study, we used item response models to test the psychometric functioning of the ASTI based on the results of the expert assignments.

### Participants

Data were collected individually from 1215 participants in Austria and Germany by trained students as part of their class work. A total of 666 participants were students (431 women, *Mdn*_*age*_ = 23, *IQR* = 5, *min* = 18, *max* = 60) and 549 were non-students (346 women, *Mdn*_*age*_ = 35, *IQR* = 23, *min* = 15, *max* = 81). Some participants failed to fill out the whole questionnaire, but the frequency of missing values per item was very low (*M* = 0.4%, *SD* = 0.27, *min* = 0, *max* = 0.7) and was not associated with external variables, suggesting that the missing values can be treated as occurring randomly.

Participants filled out a set of paper-and-pencil scales and answered demographic questions. Overall, participation took about 25 min on average. The questionnaire included the ASTI and additional scales outside the scope of this paper.

### Analytical procedures

To test the unidimensionality of the new subscales, we used an approach from the family of Rasch models. Rasch models (Rasch, 1960; for an overview see Fischer and Molenaar, 1995) and their extensions for graded response categories are very useful for testing specific hypotheses about the dimensionality of items within a scale. First, they test an assumption that is usually taken for granted when a score is computed by summing up the items of a scale: the sum score is a valid indicator of a construct only if all items measure the same latent dimension (Rasch, 1960). If, for example, some items in our scale measure non-attachment while others measure self-knowledge, and these two constructs can occur independently of each other, then summing up across all items is not informative about a person's actual construct levels. One would need to know their separate scores for the two subdimensions. Only if all items measure the same construct, the raw score is a good indicator of a person's level of that construct. The main indicators that Rasch-family models use are item parameters and person parameters, which are placed on the same latent dimension. Persons' positions on the latent dimension, their so-called person parameters, are determined by their raw scores. The higher a person's score, the more likely is the person to agree to the items of the test. Items are represented by monotonically increasing asymptotic probability curves on the same latent dimension: the probability that a person agrees to an item is dependent on the relation between the position of the item and the position of the person on the latent dimension. Each item's position is described by its item parameter, i.e., the point on the latent dimension where a person's probability of agreeing to the item is 0.50. For items with graded responses, as in the current case, parameters describe the thresholds between adjacent response categories. Here, we used the Partial Credit Model (PCM; Masters, 1982; Masters and Wright, 1997), which assumes unidimensionality of the items but does not assume that the distances between categories are equal across items.

Specifically, we used the multidimensional random coefficient multinomial logit model (MRCML model; Adams et al., 1997), a generalization of a wide class of Rasch models including the PCM. The PCM is implemented, for example, in the software ConQuest (Wu et al., 2007), in MPlus 7.4 (Muthén and Muthén, 1998–2015), and in the R-package “Test Analysis Modules” (TAM; Kiefer et al., 2015), which was used here.

The item parameters were estimated using marginal maximum likelihood estimation (MLEs) and the person parameters using weighted maximum likelihood estimation (WLEs). The item analysis procedure follows Pohl and Carstensen (2012), who outlined an approach for item analysis for use in large-scale assessment settings. We believe that this approach is also useful for smaller-scale studies.

#### Testing model fit

As explained earlier, a main goal of the study was to integrate the proposed dimensions and the experts' hypotheses concerning item fit with a psychometric investigation of the items. As shown in the section “Definition of Psychometric Hypotheses,” strong hypotheses concerning dimensionality and candidate predictors for possible item misfit were identified. Accordingly, in the psychometric analysis these predictors will be used to test for significant item misfit.

Before starting with the actual analyses, the category frequencies for each item were assessed because low frequencies can cause estimation problems. If the frequency of a response category was below 100, it was collapsed with the next category (see Pohl and Carstensen, 2012). In 11 items, the two lowest categories, and in two other items, the two highest categories were merged. In the remaining 12 items, all category frequencies were above 100. In the development of new measures, it is often a goal to have few items with very low frequencies in some response categories. With constructs like wisdom, however, which are very positively valued, few participants disagree with positively worded items, and the variance that does exist is mostly located between the middle and the highest category. If such items represent theoretically important aspects of the construct, they may well be kept as part of the scale. In the current case, low frequencies in the lowest categories were particularly typical for the SI and PG subdimensions (four items each), and removing these items would have depleted both scales of important content. In the following, we describe the analyses that were performed.

#### Person-item-maps

Person-item-maps display the distribution of the person parameters and the range of item parameters. These plots show whether any participants showed extreme response tendencies, which might lead to particularly high or low raw scores, and how the item parameters are distributed over the latent dimension. Thus, it can be examined whether the items cover the whole spectrum of the latent dimension or cluster in one part of it. If there are few items in a segment of the spectrum, the latent trait cannot be measured well in that segment.

#### Dimensionality

Up to now, the ASTI was scored as a unidimensional instrument, although the items were constructed so as to represent the subdimensions described earlier. Based on this theoretical background and the expert judgments, the five-dimensional model in Tables 3A–C was used as the starting point for the following analyses. In order to test whether the five-dimensional model fit better than the one-dimensional model, they were compared using the Bayesian information criterion (BIC; Schwarz, 1978) as recommended by, e.g., Kang and Chen (2011). Chi-square tests were also computed (see **Table 6**) however, they may be oversensitive due to the relatively large sample size. The five-dimensional model was estimated using a quasi-Monte Carlo integration (Kiefer et al., 2015) with 20,000 nodes (number of theta parameters used for the simulation). The latent correlations between the five dimensions were estimated.

#### Rasch homogeneity

Once the dimensionality of the ASTI is established, we can test the fit of the Rasch model within each subscale, analyzing several indicators of fit for each individual item. First, the assumption of Rasch homogeneity was tested by comparing the PCM against the generalized partial credit model (GPCM, Muraki, 1992), which includes different discrimination parameters across items. Only if the PCM does not fit significantly worse than the GPCM, the assumptions of the Rasch family hold for a scale and the raw score is a sufficient statistic for the person parameter.

Additionally, the expected score curves of each item were examined. **Figure 3** shows some examples of the results of this analysis. With this kind of graphical display, it is possible to examine whether the observed score curve is different from the expected curve (misfit) and whether the discrimination (slope) of an item is higher or lower than assumed by the PCM.

#### Item fit

The fit of individual items was assessed using infit and outfit statistics, i.e., the weighted and unweighted means square statistics (MNSQ; Wright and Masters, 1990; Wu, 1997), respectively. Following Wright and Linacre (1994), a range between 0.6 and 1.4 was defined as acceptable fit. Generally, a value below 1 indicates overfit (the data are too predictable) and a value above one indicates underfit of items (the data are less predictable than expected by the PCM). Overfit (e.g., too high discrimination of items) is less problematic than underfit (e.g., too high guessing probability). Thus, underfit should receive more attention in the evaluation of the items.

#### Differential item functioning (DIF)

Differential item functioning means that the pattern of response probabilities for some items differs between groups of participants. For example, gender-related DIF would mean that men are more likely to agree to some items of a scale than women. If that were the case, the scale as a whole would be measuring a somewhat different construct for men than for women. Here, DIF was tested with respect to gender (*N*_*women*_ = 777, *N*_*men*_ = 438), age (15–31 years *N* = 851, 32–81 years *N* = 364), and students vs. non-students (*N*_*students*_ = 666, *N*_*non-students*_ = 549). To assess DIF, the fit of two models was compared by means of the BIC: a main-effect model, which allows only for a main effect of the DIF variable across all items, and an interaction model, which additionally includes an interaction between the DIF variable and the items. If the interaction model fits significantly better than the main-effect model, there is a significant amount of DIF, that is, the patterns of item difficulties vary between the levels of the DIF variable. Following Pohl and Carstensen (2012) and the DIF categorization of the Educational Testing Service (Linacre, 2014) we used absolute logit differences to judge the magnitude of DIF on the item level: no relevant DIF = smaller than 0.4; slight to moderate DIF = 0.4–0.6, (C) moderate to large DIF or noteworthy for further investigations = 0.6–1, and (D) very strong DIF = larger than 1.

The experts' suggestions concerning possible DIF from Section Definition of Psychometric Hypotheses were taken into account in interpreting the results of the DIF analyses.

#### Further analyses

Descriptive statistics (*M, SD*) for each item were calculated of each dimension separately. As an index of internal consistency, we used the EAP reliability coefficient, an indicator for the PCM that is comparable with Cronbach's Alpha. The item parameters of all items (based on final scale assignment) and item intercorrelations are reported in the Appendix in the Supplementary Material to this article.

### Results

#### Person-item-map

**Figure 2:**
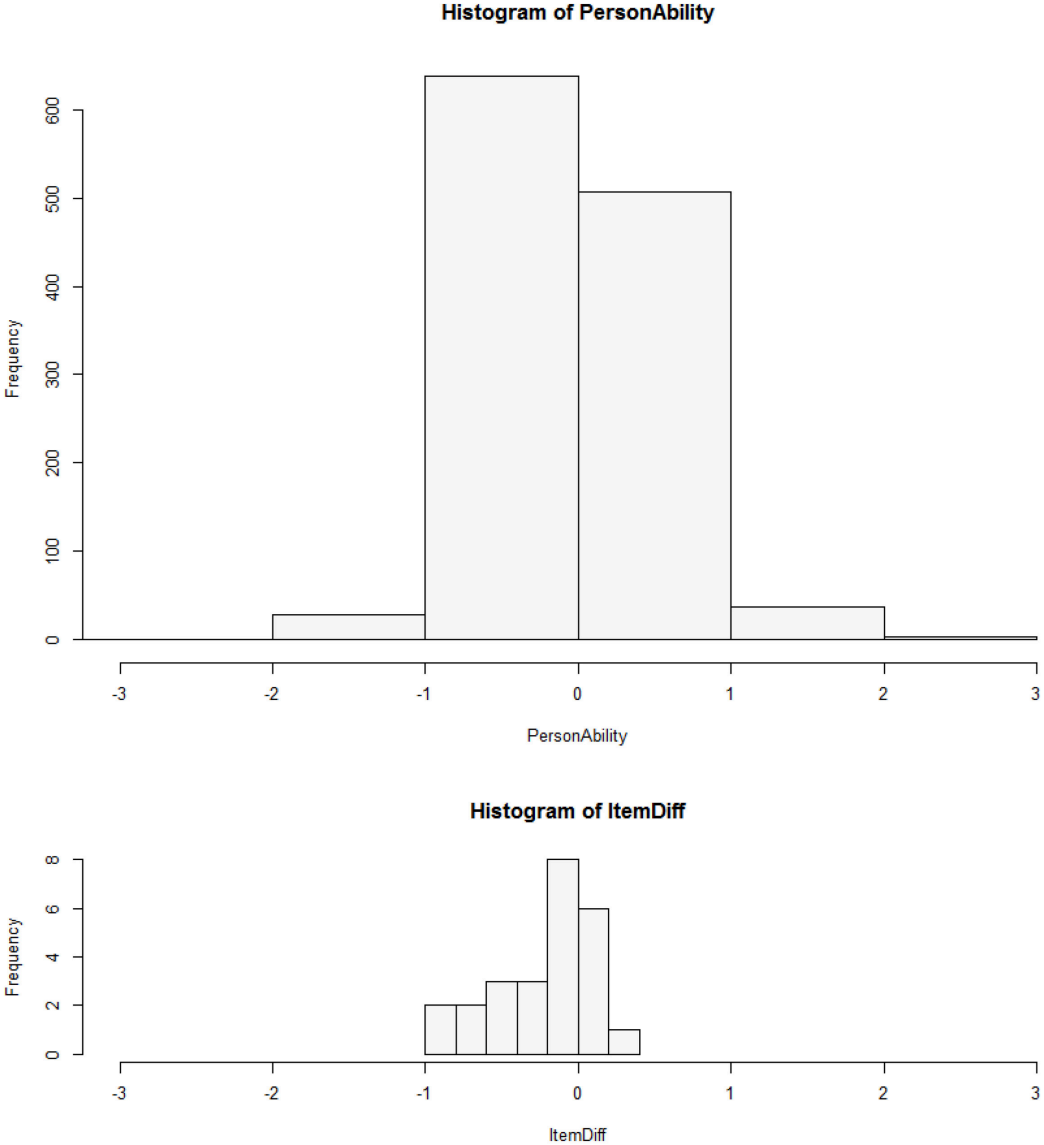
**Person-item-map**.

The person-item-map in Figure 2 shows that the item parameters mostly covered the left-hand side of the middle range of the ability parameter distribution. That is, the items were rather “easy,” i.e., participants tended to agree rather than disagree (see also the descriptive statistics in Table **6**). For the ASTI to also differentiate well among high-scoring individuals, more items should be constructed that participants are less likely to agree with. It is, however, a general problem with self-report measures of wisdom that they tend to elicit high scores, be it due to effects of social desirability or of overly positive self-evaluations (Glück et al., 2013). Performance measures of wisdom, such as the Berlin Wisdom Paradigm (Baltes and Staudinger, 2000) tend to produce far lower average levels of wisdom than self-report measures do.

#### Dimensionality

Next, four different models were estimated and compared by means of the BIC. As Table 4 shows, the GPCM (1DIM_2PL) fit the data better than the PCM (1DIM_1PL), indicating that the items differ in discrimination. Furthermore, the comparison between five-dimensional and one-dimensional models suggested that the five-dimensional models generally fit the data better than the unidimensional ones.

**Table 4:** **Comparison of the estimated IRT models**.

**Model**	**logLik**	**npar**	**BIC**
1DIM_1PL	−33300.89	63	67049.24
1DIM_2PL	−33062.81	87	66744.00
5DIM_1PL	−32871.86	77	66290.61
5DIM_2PL	−32524.25	97	65737.44
**MODEL COMPARISON OF EACH DIMENSION SEPARATELY**			
SI_1PL	−4442.59	09	8949.10
SI_2PL	−4408.00	12	8901.22
PM_1PL	−5380.55	11	10839.24
PM_2PL	−5370.76	14	10840.96
NA_1PL	−5751.28	12	11587.79
NA_2PL	−5735.62	15	11577.78
ST_1PL	−9883.43	20	19908.91
ST_2PL	−9715.91	26	19616.49
PG_1PL	−7763.56	15	15633.66
PG_2PL	−7721.62	20	15585.29

*LogLik is the log likelihood for each model, npar is the number of estimated parameters, 1PL is the PCM, and 2PL is the GPCM*.

Table 5 shows the latent correlations among the five dimensions and the EAP reliability indices, as well as Cronbach's α and the confidence interval for Cronbach's α (Fan and Thompson, 2001). EAP reliabilities were acceptable, whereas Cronbach's α were below 0.50 for PM, NA, and PG. This may be due to the relatively low variance in the item responses. The latent correlations supported the assumption of a five-dimensional structure of the ASTI. Self-knowledge and integration, peace of mind, and presence in the here-and-now and growth were quite highly correlated, which may suggest that they all represent an accepting and appreciative stance toward oneself and the experiences of one's life. Non-attachment and self-transcendence seem to be less closely related to the others (except for the correlation between non-attachment and peace of mind), possibly because they both, although in different ways, represent the individual's relationship with the external world: non-attachment describes an independence from other people and material things, and self-transcendence represents a connectedness with others and the world at large. Both may not be part of everyone's experience of inner peace. However, Table 4 showed that the five dimensional GPCM fit the data best.

**Table 5:** **Latent correlations between the five dimensions, EAP-Reliability, and Cronbachs- α incl. 95% confidence interval for each dimension**.

**Dimensions**	**SI**	**PM**	**NA**	**ST**	**PG**
SI	1	0.657	0.323	0.227	0.739
PM		1	0.591	0.323	0.721
NA			1	0.274	0.365
ST				1	0.552
PG					1
EAP-Rel.	0.692	0.626	0.508	0.668	0.660
Cronbach's α	0.642	0.449	0.426	0.636	0.384
95% Confidence	0.607;	0.396;	0.370;	0.603;	0.328;
Interval for α	0.674	0.499	0.477	0.666	0.437

#### Rasch homogeneity, item fit, and differential item functioning

Next, we assessed the items of each dimension separately. In general, the infit and outfit statistics showed no misfit of items (see Table 6). Because of the complexity of analyses, the following results are reported for each dimension separately. Table 4 shows the overall fit of the GPCM and PCM for each subdimension according to the BIC. Log likelihoods for both models are also reported, although likelihood ratio tests are likely to be somewhat oversensitive due to the large sample size.

**Table 6:** **Descriptive values (M, SD), uncentered PCM item (δ_i_), and category parameters (C_i_) estimated for each subscale separately, Itemfit Statistics (Outfit, Infit), and absolute differences (DIFF) for the three tested external variables gender, age, and group**.

**Dim**	**Item**	**Cat**.	**M**	**SD**	**δ_i_**	**SE**	**C_1_**	**SE**	**C_2_**	**SE**	**C_3_**	**SE**	**Outfit**	**Infit**	**DIFF Gender**	**DIFF Age**	**DIFF Group**
SI	10	3	1.29	0.69	−0.85	0.05	−1.98	0.09	0.27	0.07	–	–	1.108	1.097	−0.042	−0.618	−0.530
	19	3	1.28	0.65	−0.90	0.05	−2.33	0.10	0.52	0.07	–	–	0.943	0.950	−0.040	0.052	0.086
	20	3	1.24	0.69	−0.72	0.05	−1.92	0.09	0.48	0.07	–	–	0.910	0.922	0.156	0.352	0.244
	21	3	1.13	0.63	−0.43	0.05	−2.01	0.09	1.16	0.07	–	–	1.016	1.019	−0.074	0.212	0.198
PM	01	3	0.93	0.69	0.20	0.05	−0.76	0.07	1.16	0.08	–	–	1.017	1.016	−0.04	0.046	−0.012
	05	4	1.60	0.83	−0.13	0.04	−1.67	0.10	−0.21	0.06	1.47	0.09	0.968	0.967	−0.024	0.104	0.190
	09	4	1.66	0.91	−0.21	0.04	−1.37	0.10	−0.29	0.06	1.03	0.08	1.006	1.003	0.042	−0.316	−0.140
	22	3	1.04	0.67	−0.11	0.05	−1.21	0.08	1.00	0.07	–	–	1.008	1.007	0.022	0.164	−0.038
NA	03	4	1.45	0.89	0.08	0.04	−1.15	0.08	0.10	0.06	1.29	0.09	1.013	1.011	0.226	−0.076	−0.070
	06	4	1.41	0.90	0.14	0.04	−1.09	0.08	0.20	0.06	1.32	0.09	1.008	1.008	0.002	−0.042	0.002
	08	3	0.99	0.72	0.02	0.04	−0.72	0.07	0.76	0.07	–	–	0.938	0.940	0.026	0.17	0.158
	12	4	1.56	0.81	−0.11	0.04	−1.78	0.10	−0.04	0.06	1.50	0.09	1.047	1.047	−0.254	−0.052	−0.090
ST	02	4	1.69	0.91	−0.23	0.04	−1.26	0.10	−0.50	0.06	1.05	0.08	0.911	0.911	−0.140	0.07	0.042
	04	4	1.72	0.85	−0.31	0.04	−1.63	0.11	−0.58	0.06	1.26	0.08	1.044	1.036	−0.054	0.06	0.058
	07	4	1.47	0.95	0.08	0.04	−0.90	0.08	−0.05	0.06	1.21	0.09	0.883	0.889	−0.102	−0.108	−0.130
	13	3	1.27	0.73	−0.58	0.04	−1.11	0.08	−0.06	0.06	–	–	1.121	1.092	0.144	−0.29	−0.292
	16	4	1.58	0.90	−0.12	0.04	−1.34	0.09	−0.20	0.06	1.20	0.08	1.024	1.023	0.106	0.252	0.210
	24	3	0.84	0.70	0.38	0.05	−0.51	0.06	1.27	0.08	–	–	1.059	1.052	0.098	−0.012	0.032
	25	4	1.50	0.90	0.04	0.04	−1.25	0.09	−0.05	0.06	1.41	0.09	0.997	0.995	−0.054	0.028	0.080
PG	11	3	1.42	0.65	−0.92	0.05	−1.64	0.10	−0.20	0.06	–	–	0.984	0.987	−0.246	0.056	0.056
	14	4	1.65	0.88	−0.13	0.04	−1.04	0.09	−0.53	0.06	1.17	0.08	1.043	1.037	0.224	0.324	0.324
	15	3	1.38	0.64	−0.90	0.05	−1.78	0.10	−0.03	0.06	–	–	0.963	0.966	−0.062	−0.126	−0.126
	17	3	1.17	0.76	−0.31	0.04	−0.74	0.07	0.12	0.06	–	–	0.988	0.988	0.242	0.060	0.060
	18	4	1.56	0.87	−0.08	0.04	−1.33	0.09	−0.12	0.06	1.20	0.09	1.054	1.052	0.088	−0.368	−0.368
	23	3	1.20	0.72	−0.41	0.04	−1.04	0.08	0.21	0.06	–	–	0.961	0.963761	−0.246	0.052	0.052

*A negative DIFF means that the item is more difficult for women, older participants, and non-students than for men, younger people, and students, respectively*.

#### Self-knowledge and integration (SI)

**Figure 3:**
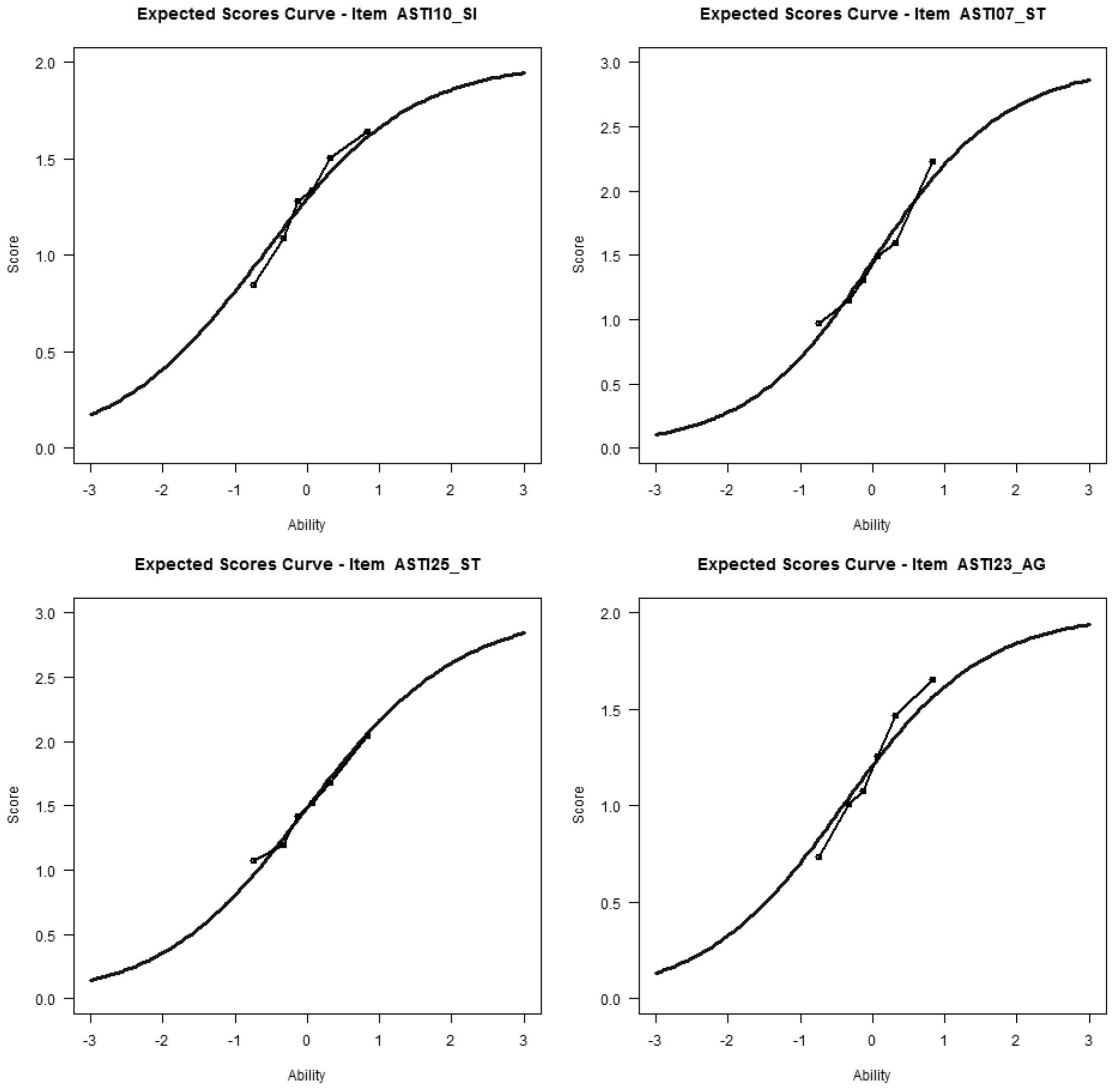
**Expected response curve or score curves**.

Table 4 shows that the GPCM fit the data better than the PCM. The score curves suggest that, generally, the observed slopes were steeper than expected; the observed slope of item 10 also showed small deviations from the expected slope (see Figure 3). When item 10 was excluded, the difference in fit between the GPCM and PCM became quite negligible (PCM: log likelihood = −3277.90, npar = 7, BIC = 6605.50; GPCM: log likelihood = −3269.56, npar = 9, BIC = 6603.03). Therefore, the PCM was considered to fit the scale sufficiently well when item 10 was excluded. As explained earlier, DIF was assessed with respect to gender, age, and professional group. Men's person parameters (*SD* = 1.21, *d* = 0.30) were, on average, 0.27 logits higher than women's (indicating that men were higher in self-knowledge and integration than women); there were no significant main effects for age (*M*_*difference*_ = 0.06, *SD* = 1.23, *d* = 0.05) or students vs. non-students (*M*_*difference*_ = 0.01, *SD* = 1.23, *d* = 0.01). However, the model comparisons in Table 7 indicated DIF for age and group. Only item 10 (“I have a good sense of humor about myself”) showed considerable DIF: it was more often agreed to by younger participants and students than by older participants and non-students, respectively. Note that Item 10 had not received an unequivocal assignment by the experts either (see Table 3A). In addition, item 20 (“I am accepting of myself, including my faults”) was more often agreed to by older participants and non-students than by younger people and students. However, the magnitude of DIF was small and could therefore be ignored. When the analyses were repeated excluding item 10, the PCM fit the data well and there was no considerable DIF for any item.

**Table 7:** **Comparison of the main-model against the DIF interaction-model**.

**Dim**	**Var**	**Main-model**			**DIF interaction-model**		
		**logLik**	**npar**	**BIC**	**logLik**	**npar**	**BIC**
SI	Gender	−4438.3	10	8947.62	−4436.87	13	8966.08
	Age	−4442.48	10	8955.99	−4420.46	13	8933.25
	Group	−4442.59	10	8956.20	4424.60	13	8941.53
EM	Gender	−5362.98	13	10818.30	−5362.68	16	10839.00
	Age	−5378.62	13	10849.56	−5368.48	16	10850.61
	Group	−5378.51	13	10849.34	−5373.51	16	10860.66
NA	Gender	−5749.97	13	11592.27	−5740.28	16	11594.20
	Age	−5742.59	13	11577.52	−5740.19	16	11594.01
	Group	−5742.27	13	11576.87	−5739.46	16	11592.57
ST	Gender	−9864.67	21	19878.50	−9858.77	27	19909.31
	Age	−9871.33	21	19891.81	−9860.06	27	19911.89
	Group	−9880.66	21	19910.47	−9868.11	27	19927.99
CO	Gender	−7762.27	17	15645.29	−7745.62	22	15647.49
	Age	−7762.64	17	15646.03	−7741.21	22	15638.67
	Group	−7756.91	17	15634.55	−7743.28	22	15642.81

*LogLik is the log likelihood for each model, npar is the number of estimated parameters*.

#### Peace of mind (PM)

Table 4 shows that the difference in BIC between the GPCM and PCM is again negligible, suggesting acceptable fit of the PCM for this scale. The DIF analyses indicated that women (*M*_*difference*_ = 0.334, *SD* = 0.622, *d* = 0.54), older participants (*M*_*difference*_ = 0.152, *SD* = 0.644, *d* = 0.24), and non-students (*M*_*difference*_ = 0.114, *SD* = 0.643, *d* = 0.18) received higher person parameters than men, younger participants, and students, respectively, but there was no differential item functioning (see Tables 6, 7).

#### Non-attachment (NA)

The GPCM fit the scale better than the PCM, but again, the difference in BIC was small and the score curves showed good agreement between expected and observed response curves and slopes (see Table 4). Thus, the PCM was preferred. Again, there was no indication of DIF (see Tables 6, 7), but women (*M*_*difference*_ = 0.076, *SD* = 0.578, *d* = 0.13), older participants (*M*_*difference*_ = 0.240, *SD* = 0.656, *d* = 0.43), and non-students (*M*_*difference*_ = 0.222, *SD* = 0.566, *d* = 0.39) received higher person parameters than men, younger participants, and students, respectively.

#### Self-transcendence (ST)

Table 4 shows that the GPCM fit the data substantially better than the PCM. It is somewhat unclear, however, what causes the difference in fit, as the two examples of score curves in Figure 3 represent the general result for all items of this scale, indicating no substantial underfit or overfit. It seems important to reanalyze the self-transcendence scale with new data. The DIF analyses indicated that women (*M*_*difference*_ = 0.292, *SD* = 0.661, *d* = 0.60), older participants (*M*_*difference*_ = 0.248, *SD* = 0.668, *d* = 0.37), and non-students (*M*_*difference*_ = 0.106, *SD* = 0.676, *d* = 0.16), again received higher person parameters than men, younger participants, and students, respectively. As Tables 6, 7 show, no substantial DIF was found for this subscale.

#### Presence in the here-and-now and growth (PG)

Table 4 indicates that the GPCM fit the data slightly better than the PCM. The score curves (for an example see Figure 3, left below) showed that the observed slopes were slightly higher than the expected slopes. Non-students (*M*_*difference*_ = 0.162, *SD* = 0.450, *d* = 0.36) had lower person parameters than students; there were no significant differences for gender or age (gender: *M*_*difference*_ = 0.04, *SD* = 0.455, *d* = 0.09; age group: *M*_*difference*_ = 0.066, *SD* = 0.458, *d* = 0.14). Item 14 (“I am not often fearful”) had a small amount of DIF for all three group variables, i.e., it was more difficult to agree to for men, younger participants, and students than for women, elderly people, and non-students, respectively. It was also the only negative item (see Table 3C) in the subdimension. When item 14 was excluded, the difference in fit between the PCM and GPCM was markedly reduced (PCM: log likelihood = −6241.90, npar = 12, BIC = 12569.04; GPCM: log likelihood = −6208.536, npar = 16, BIC = 12530.71). This subscale should also be reanalyzed once new data are available. A re-analysis without item 14 showed that the PCM fit the data well.

## Discussion

In the following, we first discuss the methodological implications of our research, and then, its substantive implications concerning the use of the ASTI to measure wisdom.

### Methodological conclusions: a comprehensive approach for evaluating content validity

This paper introduced the CSS procedure for evaluating content validity and discussed its advantages for the theory-based evaluation of scale items. In our experience, the method provides highly interesting practical and theoretical insights into target constructs. It does not only allow for evaluating and validating existing instruments and for improving the operationalization of a target construct, but it also offers advantages for constructing new items for existing instruments or even for developing whole new instruments. The procedure can be applied in all subdisciplines of psychology and other fields, wherever the goal is to measure specific constructs. In addition, it does not matter which kinds of items (e.g., questions, vignettes) and response formats (e.g., dichotomous, graded, open-ended) are used. The in-depth examination of the target construct is likely to increase the validity of any assessment.

We propose to follow certain quality criteria in studies using our approach. First, to optimize replicability, all steps should be carefully documented. A detailed documentation of procedures increases the validity of the study, irrespective of whether the data collection is more quantitative (as in the present study) or more qualitative (e.g., focus group discussions as in Castel et al., 2008). Second, the selection of experts is obviously crucial. Objectivity may be compromised if the group of experts is too homogeneous (e.g., if they are all from one research group) or too small. The instructions that the experts receive also need to be carefully written so as to avoid inducing any biases. Third, it is important that the expert judgments are complemented by actual data collected from a sample representative of the actual target population. Our experience is that the data are often astonishingly consistent with the expert ratings; however, experts may also be wrong occasionally, for example, if they assume more complex interpretations of item content than the actual participants use. As we have demonstrated here, item response models may be particularly suited for testing hypotheses about individual items, but factor-analytic approaches are also very useful for testing hypotheses about the structural relationships between subscales. For example, it would be worthwhile to test the current data for a bi-factor structure, i.e., a combination of a significant common factor with subscale-specific factors. Next steps in our work will include the comparison of these different methods of data analysis. Another important future goal is the definition of a quantitative content-validity index based on the current method.

### Substantive conclusions: measuring self-transcendence using the ASTI

In addition to utilizing the ASTI to demonstrate our approach, we believe that we have gained important insights about the ASTI, as well as about self-transcendence in general, from this study. Through the exercise of assigning and reassigning the items to the dimensions of the construct and discussing the contradictions and difficulties we encountered, we gained a far deeper understanding of the measured itself.

In general, the analyses demonstrated the importance of constructing more “difficult” items, i.e., items with a lower level of agreement. This is a general issue with self-report wisdom scales (see Glück et al., 2013): items representing core capacities of wisdom, such as being able to consider different perspectives, to be compassionate even with strangers, or to integrate conflicting aspects of the self may sound appealing even to individuals who are rather low in these capacities in real life. In fact, wiser individuals may even be more critical of themselves and thus less likely to rate themselves highly than other people are (Aldwin, 2009; Glück et al., 2013). Some of the ASTI items nicely evade this problem by being difficult to understand for individuals who have not achieved the respective levels of self-transcendence. For example, the item “Whatever I do to others, I do to myself” regularly leaves our student participants dumbfounded. The positive German version of this item had the lowest mean, i.e., the lowest agreement frequencies, of all items in the scale. It may be worthwhile to try to construct more items of this kind.

For now, we have identified five subdimensions that include the 24 positive items (in German, 25) of the ASTI. The 10 negative items measuring alienation were not included in this analysis, as negative items tend to be difficult to assign to the same dimension as positive items. We recommend to leave them in the questionnaire in order to increase the range of item content, but to exclude them from score computations. In further applications of the ASTI, should the five subdimensions be scored separately or should the total score be used? Strong advocates of the Rasch model would certainly argue that using the total score across the subdimensions amounts to mixing apples and oranges. However, other self-report scales of wisdom such as the 3D-WS (Ardelt, 2003) or the SAWS (Webster, 2007) measure several dimensions of wisdom that are conceptually and empirically related to about the same degree as the subdimensions of the ASTI we have identified here. Both these authors suggest to use the mean across the subdimensions as an indicator of wisdom and to consider only individuals as wise who have a high mean, i.e., they score high in all subdimensions. The same may be a good idea here: for an individual to be considered as highly wise (in the sense of self-transcendence), he or she would need to have high scores in all five subdimensions, as all of them are considered as relevant components of wisdom. For individuals with lower means, we recommend to consider their profile across the subdimensions rather than compute a single score.

### The subdimensions of the ASTI

In the following, we describe the final subdimensions of the ASTI that resulted from our analysis and relate them to the theoretical background, thus completing point (8) “Final Definition of the Latent Construct” in the process. The subdimensions are ordered so as to represent a possible developmental order as suggested by Levenson et al. (2005), with self-knowledge and integration as well as non-attachment preceding presence in the here-and-now and peace of mind, and self-transcendence being the last (and probably rarest) stage. It is important to note that in addition to producing valid and reliable subdimensions, the CSS procedure has also led us to conceptually redefine some of the subdimensions so as to better differentiate them (for example, independence of external sources of well-being was originally included in the definitions of both non-attachment and self-transcendence). We first give definitions for all subdimensions and then discuss their relationships to each other and to age and gender.

#### Self-knowledge and integration

The first subdimension includes items that were originally intended to measure Curnow's (1999) separate dimensions of self-knowledge and integration. It includes items that refer to broad and deep knowledge about as well as acceptance of all aspects of one's own self, including ambivalent or undesirable ones. Thus, the distinction between being aware of certain aspects of the self and accepting them was not supported empirically. The idea that self-knowledge and the acceptance of all aspects of the self is key to wisdom can be found in Erikson's (1980) idea of integrity, i.e., late-life acceptance of one's life as it has been lived (see also Beaumont, 2009).

Individuals high in this dimension of the ASTI are aware of the different, sometimes contradictory, facets of their self and their life, and they are able to accept all sides of their personality and integrate the different facets of their life. If the item “I have a good sense of humor about myself,” which was somewhat equivocal among the experts and showed differential item functioning in the quantitative analyses, is excluded, the subdimension includes only three items. Therefore, it seems advisable to add new items that refer to self-knowledge as well as items that differentiate between different kinds of integration (e.g., integration of self aspects, life contexts, and feelings). With a higher number of items, the distinction between knowing and accepting aspects of one's self might also receive more empirical support.

#### Non-attachment

Non-attachment describes an individual's awareness of the fundamental independence of his or her internal self of external possessions or evaluations: non-attached individuals' self-esteem is not dependent on how others think about them or how many friends they have. The scale comprises four items concerning the individual's independence of external things, such as other people's opinions, a busy social life, or material possessions. It is important to note that non-attachment does not mean that people are not committed to others or to important issues in their current world; the main point is that they do not depend on external sources for self-enhancement. The fact that they are not affected by other people's judgments enables them to lead the life that is right for them and accept others non-judgmentally. Like other ideas originating from Buddhism, non-attachment as a path to mental health is currently receiving some attention in clinical psychology (Shonin et al., 2014), but it has not yet been investigated in psychological wisdom research.

#### Presence in the here-and-now and growth

Individuals high in this dimension, which was not part of Curnow's (1999) original conception, are able to live in the moment and enjoy the good times in their life without clinging to them, because they know that everything changes and that change may also foster growth. The items of this subdimension describe individuals who are able to live life mindfully in any given moment: they find joy in their life and in what they are doing. They are aware that things are always changing, oriented toward learning from others, and aware that they have grown through losses, and they have accepted the finitude of life. In a different study, we have found that many wisdom nominees report gratitude for the difficult experiences of their lives, i.e., they appreciate the processes of learning and growth triggered by such events (Bluck and Glück, 2004; König and Glück, 2014).

#### Peace of mind

Tranquility is a characteristic that many laypeople associate with wisdom (Bluck and Glück, 2005); the related construct of emotion regulation has been proposed to be both a component of wisdom (Webster, 2003, 2007) and a developmental resource for wisdom (Glück and Bluck, 2013). This subdimension of the ASTI describes individuals who are able to maintain their tranquility in situations where others would get angry or upset, and are at peace with the fundamental impermanence of things in life.

#### Self-transcendence

Highly self-transcendent individuals feel that the boundaries between them and others, even humanity at large, are permeable. They feel related to past and future generations, all human beings, and nature. As they do not need to utilize social relationships to enhance their sense of self, they are able to love and accept other individuals as they are. As Levenson et al. (2005) argued, self-transcendence may be at the core of wisdom (cf. Tornstam, 1994).

### Latent correlations between the five dimensions

There were relatively high latent correlations (around 0.70) between the subdimensions of self-knowledge and integration, peace of mind, and presence in the here-and-now and growth, all of which seem to describe an accepting and appreciative stance toward oneself and one's life. For some purposes, it may make sense to average across these three subdimensions, as their discriminant validity may be limited. At the same time, the manifest correlations between these three subscales are markedly lower than the latent ones *r* (SI-PM) = 0.36, *r* (SI-PG) = 0.41, *r* (PM-PG) = 0.35; thus, the subscales may well be differentially related to other constructs. Therefore, we recommend to treat them separately for most research purposes. Non-attachment and self-transcendence were less closely related to the others and to each other, perhaps because they represent two important and somewhat contrary aspects of wise individuals' relationship with the external world: independence of one's self from external sources and a deep connectedness to others and the world. Our findings suggest that each of these states can exist without the other, and both can be present in an individual without the peace of mind that comes with self-integration and living in the present. A truly wise individual, however, would show high levels of all of these aspects.

### Meaningful individual differences

The individual differences in our data (see “Differential Item Functioning”) were largely consistent with the literature. It is important to first note that our sample is not well-suited for analyzing older age: we were able to compare only two age groups roughly corresponding to adolescence and young adulthood on the one hand (15–31 years, *N* = 851; including many students) and early middle to older adulthood on the other hand (32–81 years, *N* = 364). A further differentiation among the “older” adults was not possible because only 3.3% of the sample were 60 years old or older. Comparing the two age groups, we found meaningful differences for two of the five dimensions. People older than 31 achieved higher scores in non-attachment and self-transcendence than adolescents and young adults. In another study with a mostly older sample, we found no correlation between the ASTI and age (Glück et al., 2013). As has been shown for cognitive aspects of wisdom (Pasupathi et al., 2001; Staudinger and Pasupathi, 2003), some facets of wisdom may develop in young adulthood and then stay stable into old age. The other three subdimensions, which represent an appreciative and accepting stance toward life, do not seem to be dependent on age.

Gender differences were found, interestingly, for four of the five subdimensions. Men had higher scores than women in self-knowledge and integration. This finding may suggest that men indeed know and accept themselves more than women do or that women actually tend to be more self-reflective and self-critical. In any case, the effect was small and needs further investigation. In the subdimensions peace of mind, non-attachment, and self-transcendence, women scored higher than men. These findings may, however, be partly determined by societal expectations for women to be less self-centered and more caring than men, which does not necessarily imply true self-transcendence. Thus, the limitations of self-report measures remain somewhat present even in carefully constructed scales like the ASTI.

## Conclusion

In sum, we suggest that researchers using the ASTI may gain significant information if they use separate scores for the subdimensions we have identified in addition to, or instead of, the total score. The self-transcendence subdimension may be the purest indicator of actual self-transcendence. Whether the other subdimensions represent important preconditions, correlates, or even outcomes of self-transcendence is largely an empirical issue to be addressed in the future, which may tell us more about the development of wisdom.

## Ethics statement

No formal approval was applied for as the guidelines of the local Ethics Committee specify that the type of survey study we performed does not require such approval. All participants filled out an informed-consent form and agreed that their data are used for scientific purposes. No vulnerable populations were involved in this study.

## Author contributions

All three authors meet the four criteria for authorship required in the author guidelines. Each author's main tasks were as follows. IK: Development and application of the CSS procedure, expert in the first part of study, data analyses, writing the paper. JG: Discussion partner for the CSS procedure, expert in the first part of study, writing the parts concerning the topic of wisdom (background and results), editing of the manuscript. ML: Construction and provision of the revised (and as yet unpublished) ASTI, discussion of the translation of the items, expert in the first part of the study.

## Funding

This research was partly funded by the Austrian Science Fund FWF (grant nr. P25425, PI: JG).

### Conflict of interest statement

The authors declare that the research was conducted in the absence of any commercial or financial relationships that could be construed as a potential conflict of interest.
