# Supplementary material for: What Do You Think You Are Measuring? A Mixed-Methods Procedure for Assessing the Content Validity of Test Items and Theory-Based Scaling
Source: Front Psychol. 2017 Feb 21;8:126. doi: 10.3389/fpsyg.2017.00126 (PMC5318383; doi:10.3389/fpsyg.2017.00126)
Supplement: Supplementary file 1 [file DataSheet1.docx]

**Appendix**

Item parameters and item intercorrelations.

|  | Item par. | | SI | | | PM | | | | NA | | | | ST | | | | | | | PG | | | | | |
| --- | --- | --- | --- | --- | --- | --- | --- | --- | --- | --- | --- | --- | --- | --- | --- | --- | --- | --- | --- | --- | --- | --- | --- | --- | --- | --- |
|  | (SE) | I19 | | I20 | I21 | I01 | I05 | I09 | I22 | I03 | I06 | I08 | I12 | I02 | I04 | I07 | I13 | I16 | I24 | I25 | I11 | I14 | I15 | I17 | I18 | I23 |
| I10 | -.85  (.05) | .24 | | .29 | .18 | .08 | .17 | .10 | .16 | .15 | .05 | .05 | .09 | .01 | .06 | .01 | .08 | .09 | .11 | .06 | .24 | .13 | .16 | .16 | .05 | .18 |
| I19 | -.90  (.05) |  | | .45 | .32 | .11 | .24 | .07 | .16 | .10 | .07 | .01 | .04 | .11 | .08 | .03 | -.05 | .07 | .13 | .05 | .26 | .10 | .12 | .19 | -.05 | .12 |
| I20 | -.72  (.05) |  | |  | .35 | .12 | .28 | .09 | .27 | .20 | .06 | .09 | .05 | .07 | .09 | -.02 | .02 | .07 | .13 | .07 | .37 | .16 | .19 | .24 | -.08 | .20 |
| I21 | -.43  (.05) |  | |  |  | .08 | .22 | .07 | .25 | .08 | .06 | .05 | .00 | .10 | .09 | .02 | -.01 | .11 | .11 | .02 | .32 | .14 | .12 | .16 | -.10 | .15 |
| I01 | .20  (.05) |  | |  |  |  | .14 | .14 | .10 | .05 | .07 | .11 | .11 | .19 | .10 | .14 | .05 | .21 | .06 | .07 | .09 | .06 | .17 | .11 | .10 | .07 |
| I05 | -.13  (.04) |  | |  |  |  |  | .25 | .25 | .19 | .13 | .20 | .02 | .08 | .05 | .03 | .03 | .05 | .06 | .03 | .26 | .19 | .09 | .14 | -.03 | .09 |
| I09 | -.21  (.04) |  | |  |  |  |  |  | .12 | .10 | .06 | .13 | .10 | .04 | -.02 | .01 | .06 | .05 | .02 | .03 | .05 | .12 | .03 | .11 | .01 | .00 |
| I22 | -.11  (.05) |  | |  |  |  |  |  |  | .11 | .07 | .15 | .11 | .06 | .06 | .04 | .05 | .09 | .09 | .06 | .22 | .16 | .15 | .35 | .02 | .23 |
| I03 | .08  (.04) |  | |  |  |  |  |  |  |  | .16 | .26 | .10 | .00 | -.02 | -.02 | -.04 | .07 | .04 | .00 | .10 | .16 | .04 | .08 | -.01 | .08 |
| I06 | .14  (.04) |  | |  |  |  |  |  |  |  |  | .31 | .03 | .00 | .01 | .02 | .01 | .07 | .10 | .01 | .01 | .08 | .00 | .08 | .02 | .06 |
| I08 | .02  (.04) |  | |  |  |  |  |  |  |  |  |  | .15 | .08 | .00 | .08 | .08 | .13 | .01 | .02 | .00 | .07 | -.02 | .09 | .03 | .05 |
| I12 | -.11  (.04) |  | |  |  |  |  |  |  |  |  |  |  | .07 | .08 | .10 | .18 | .17 | .03 | .15 | .03 | .02 | .10 | .06 | .05 | .07 |
| I02 | -.23  (.04) |  | |  |  |  |  |  |  |  |  |  |  |  | .22 | .65 | .12 | .25 | .12 | .18 | .15 | .03 | .16 | .02 | .04 | .12 |
| I04 | -.31  (.04) |  | |  |  |  |  |  |  |  |  |  |  |  |  | .26 | .10 | .23 | .05 | .13 | .18 | .04 | .16 | .07 | .06 | .11 |
| I07 | .08  (.04) |  | |  |  |  |  |  |  |  |  |  |  |  |  |  | .12 | .26 | .09 | .22 | .10 | .01 | .11 | .02 | .07 | .12 |
| I13 | -.58  (.04) |  | |  |  |  |  |  |  |  |  |  |  |  |  |  |  | .09 | .03 | .20 | .05 | .00 | .15 | .03 | .08 | .10 |
| I16 | -.12  (.04) |  | |  |  |  |  |  |  |  |  |  |  |  |  |  |  |  | .11 | .15 | .12 | .06 | .12 | .12 | .08 | .16 |
| I24 | .38  (.05) |  | |  |  |  |  |  |  |  |  |  |  |  |  |  |  |  |  | .37 | .15 | .04 | .12 | .01 | .08 | .24 |
| I25 | .04  (.04) |  | |  |  |  |  |  |  |  |  |  |  |  |  |  |  |  |  |  | .07 | .05 | .14 | .05 | .09 | .18 |
| I11 | -.92  (.05) |  | |  |  |  |  |  |  |  |  |  |  |  |  |  |  |  |  |  |  | .09 | .23 | .12 | -.07 | .20 |
| I14 | -.13  (.04) |  | |  |  |  |  |  |  |  |  |  |  |  |  |  |  |  |  |  |  |  | .04 | .12 | .00 | .00 |
| I15 | -.90  (.05) |  | |  |  |  |  |  |  |  |  |  |  |  |  |  |  |  |  |  |  |  |  | .13 | .09 | .21 |
| I17 | -.31  (.04) |  | |  |  |  |  |  |  |  |  |  |  |  |  |  |  |  |  |  |  |  |  |  | .07 | .13 |
| I18 | -.08  (.04) |  | |  |  |  |  |  |  |  |  |  |  |  |  |  |  |  |  |  |  |  |  |  |  | .12 |
| I23 | -.41  (.04) |  | |  |  |  |  |  |  |  |  |  |  |  |  |  |  |  |  |  |  |  |  |  |  |  |
